# Weighing patient attributes in antibiotic prescribing for upper respiratory tract infections: A discrete choice experiment on primary care physicians in Hubei Province, China

**DOI:** 10.3389/fpubh.2022.1008217

**Published:** 2022-12-20

**Authors:** Tianqin Xue, Chaojie Liu, Zhuoxian Li, Junjie Liu, Yuqing Tang

**Affiliations:** ^1^School of Medicine and Health Management, Tongji Medical College, Huazhong University of Science and Technology, Wuhan, Hubei, China; ^2^Key Research Institute of Humanities and Social Sciences of Hubei Provincial Department of Education, Wuhan, Hubei, China; ^3^School of Psychology and Public Health, La Trobe University, Melbourne, VIC, Australia; ^4^Medical Record Management Department, Yueyang Maternal and Child Health-Care Hospital, Yueyang, Hunan, China; ^5^School of Statistics and Mathematics, Central University of Finance and Economics, Beijing, China

**Keywords:** antibiotic prescribing, discrete choice experiment, upper respiratory tract infections (URTIs), primary care, China

## Abstract

**Objectives:**

This study aimed to determine how primary care physicians weigh intervenable patient attributes in their decisions of antibiotic prescribing for upper respiratory tract infections (URTIs).

**Methods:**

A discrete choice experiment (DCE) was conducted on 386 primary care physicians selected through a stratified cluster sampling strategy in Hubei province, China. The patient attributes tested in the DCE were identified through semi-structured interviews with 13 primary care physicians, while the choice scenarios were determined by a D-efficient design with a zero prior parameter value. Conditional logit models (CL) and mixed logit models (MXL) were established to determine the preference of the study participants in antibiotic prescribing for URTI patients with various attributes. Relative importance (RI) was calculated to reflect the influence of each attribute.

**Results:**

In addition to age and duration of symptoms, the interventionable patient attributes were also considered by the primary care physicians in their antibiotic prescribing decisions. They preferred to prescribe antibiotics for URTI patients with difficulties to schedule a follow-up appointment (*p* < 0.001) and for those without a clear indication of refusal to antibiotics (*p* < 0.001). Patient request for antibiotics had an RI ranging from 15.2 to 16.3%, compared with 5.1–5.4% for easiness of follow-up appointment. The influence of these two interventionable patient attributes was most profound in the antibiotic prescribing decisions for patients aged between 60 and 75 years as indicated by their interaction effects with age (β = 0.69 for request for antibiotics, *p* < 0.01; β = −1.2 for easiness of follow-up, *p* < 0.001).

**Conclusion:**

Reducing patient pressure and improving accessibility and continuity of care may help primary care physicians make rational antibiotic prescribing decisions for URTIs.

## 1. Introduction

Antibiotic abuse has been identified as a major public health challenge (1). Globally, antibiotic consumption reached 40.1 billion defined daily doses (DDDs) in 2018, increasing from 9.8 DDDs per 1,000 inhabitants per day in 2000 to 14.3 in 2018 (2). Higher levels of antibiotic consumption are associated with increased prevalence of antibiotic resistance (3). In combination with a lack of new development of effective antibiotics, this can lead to increased morbidity and mortality of infectious diseases (4). It was estimated that 4.95 million people died from a condition that was associated with drug-resistant infections in 2019, while antibiotic resistance contributed to 1.27 million deaths directly (5). The cumulative economic loss resulting from antibiotic resistance could amount to US$2.9 trillion by 2050 (6).

The majority of antibiotic prescriptions took place in primary care, and a large proportion of these prescriptions were inappropriate (7). Primary care accounts for around 80% of all antibiotic prescriptions in the National Health Service of the UK, which is likely to be the same worldwide (8). The World Health Organization (WHO) recommends that the proportion of patients receiving antibiotics in outpatient settings should be <30% (9). However, a meta-analysis of the studies in low- and middle-income countries shows that the pooled proportion of primary care patients who were given antibiotic prescriptions has exceeded 50% (10). Several large-sample studies in low- and middle-income countries showed that more than 60% of antibiotic prescribing in primary care is inappropriate (11–13).

China is the world's second largest consumer of antibiotics (14). Between 2011 and 2018, the average antibiotic consumption per capita in China increased by 39.6% (15). A nationwide study in mainland China showed that 45% of the outpatient antibiotic prescriptions in secondary and tertiary hospitals over the period from 2014 to 2018 were inappropriate (16). The proportion of inappropriate antibiotic prescriptions in primary care is likely to exceed 50% during 2009 and 2011, despite the fact that over half of all primary care prescriptions contain antibiotics (12). Although recent publications reported a decreasing trend of antibiotic use in primary care in China, the proportion of inappropriate antibiotic prescribing (e.g., excessive use of broad spectrum and injectable antibiotic products) is increasing (17).

Antibiotics are frequently prescribed for treating upper respiratory tract infections (URTIs) in primary care (18), despite strong discouragement in clinical guidelines (19). In China, more than 40% of URTI patients are prescribed with antibiotics (20). A previous study found that the vast majority of cases of colds (78.0%) and acute bronchitis (93.5%) were treated with antibiotics in primary care in China (12).

The inappropriate prescribing decision is shaped by many factors. Internationally, extensive studies have been undertaken to explore the underlying complex causes of variations in antibiotic prescribing. Blaser et al. offered a typology categorizing the determinants of antibiotic prescribing decisions, which include the characteristics of prescribers, public understanding of antibiotic needs, patient expectation and pressure, specific social and economic interactions between prescribers and patients, financial incentives, and cultural factors (21). All of these factors play a role in antibiotic prescribing for URTIs in primary care (22). In China, patient concerns of health consequences and requests for antibiotics, a lack of knowledge and the low competency of primary care physicians in managing diagnostic uncertainty, time constraints, poor communication, and perverse financial incentives have been identified as major contributors to the over-use of antibiotics in primary care (23–25).

There has been a strong call for patient-centeredness in primary care. However, our knowledge of patient expectations and how primary care physicians respond to patient requests is very limited (26). A study of general practitioners in Australia showed that antibiotic prescribing decisions are shaped by patient life events and expectations (27). In China, antibiotic products are highly affordable thanks to the increased wealth, high coverage of insurance subsidies (>95%), and low price of prescribed medicines (28). This has led to higher antibiotic prescribing rates in regions with low socioeconomic development in comparison with their more developed counterparts (29). Empirical evidence shows that consumers in China are likely to hold the misbelief that antibiotics are anti-inflammatory (30). Failing to meet patient expectations can lead to patient complaints and ultimately loss of patients to other providers (31). In a study in Hong Kong, Lam et al. found that the patients with a regular physician were nearly twice as likely to report antibiotic use for URTIs as those without one (32).

This study aimed to address the gap in the literature by determining the antibiotic prescribing preference of primary care physicians for URTIs through a discrete choice experiment (DCE) in China. We hypothesized that both clinical and non-clinical attributes play a role in the decisions of antibiotic prescribing for URTIs in primary care. Some of the patient attributes are deemed non-clinical and interventionable. Internationally, behavioral and financial interventions on consumers have become one of the major strategies to contain antibiotic consumption in the community (33).

## 2. Methods

### 2.1. Study setting

This study was conducted in Hubei province in central China, which covers a geographic catchment of 185,900 km^2^. In 2018, Hubei recorded 59.17 million residents. Its GDP (39,367 million CNY) accounted for 4.7% of the national total, resulting in a per capita GDP (66,616 CNY) slightly higher than the national average (64,644 CNY). On average, each urban and rural resident in Hubei had a disposable income 34,455 and 14,978 CNY, respectively, in 2018.

The primary care sector in China is dominated by urban community health centers and rural township health centers. In 2018, Hubei had 354 community health centers and 1,139 township health centers, employing 8,376 and 28,370 registered (assistant) physicians, respectively. They received 70.32 million patient visits, contributing to 36.2% of all outpatient visits in Hubei (34).

### 2.2. Ethical approval

This study was approved by the Research Ethics Committee of Tongji Medical College, Huazhong University of Science and Technology (No. IORG0003571). Oral informed consent was obtained from each participant prior to data collection.

### 2.3. Study design

#### 2.3.1. Identification of tested attributes and levels

We followed the requirements recommended by Coast et al. for attribute development (35). A literature review was first conducted and then an interview guide was developed. Semi-structured in-depth interviews were conducted with 13 primary care physicians (Appendix 1) conveniently selected from 10 primary care institutions in Luohe municipality in Henan, a neighboring province of Hubei in central China. Data were collected between January and March 2019. The interview questions were centered on the decision-making process of antibiotic prescribing for URTIs in primary care settings. Each interview lasted for 30–45 min.

All interviews were audio recorded and transcribed verbatim. Two researchers (XT and LZ) coded the data simultaneously using Nvivo software version 11. An iterative process of reflection and discussion was adopted until agreement was reached between the two researchers. Disputes on the coding, if any, were resolved by the moderation of a third researcher (TY).

The coding followed the steps of inductive content analysis as described by Graneheim and Lunndman (36). First, the meanings of the phrases relevant to antibiotic prescribing were extracted and labeled with a code. Then, the various codes were compared and sorted into categories based on their differences and similarities in meaning. Finally, the latent contents of the relevant categories were formulated into themes relating to patient attributes associated with antibiotic prescribing for URTIs. A total of 14 categories under six themes emerged (Table 1).

**Table 1:** Patient attributes associated with antibiotic prescribing for upper respiratory tract infections in primary care^*^.

**Theme**	**Categories**	**Brief explanation**
Health (antibiotic) literacy	Request for antibiotics	Expression of patients wanting or not wanting antibiotics
	Perceived value of antibiotics	Patient perception of the value of antibiotics
Economic status	Affordability of medicines	Household income and expense of medicines patients willing to pay
	Financial burden of medicines	Out-of-pocket payment for medicines
Patient-physician relationship	Familiarity	The degree of familiarity between patients and primary care physicians
	Patient satisfaction	Patient feeling, attitudes and satisfaction toward primary care physicians
	Patient trust	Patient trust in primary care physician and acceptance of their assessment
Prescription filling	Reasons for not filling prescriptions in primary care facilities	One or a combination of several reasons: complicated medical procedure; high price of medicines; inadequate coverage of essential medicines in primary care
	Perceived risks of not filling prescriptions in primary care facilities	Patient concerns of poor quality of products, low compliance with instructions of medicine usage, poor management, and profit-driven behaviors
Response to treatment	Existing treatment regime	Whether patients have already been treated with antibiotics and/or other procedures
	Referral	Whether patients will be referred to a higher level of medical institutions for further investigation/treatment
	Follow-up visit	Whether patients feel it is easy to return for a visit
Predisposing factor	Clinical characteristics	Seriousness and duration of symptoms, chief complaints, type of infection, immunity, allergy, comorbidity, and family history
	Demographic characteristics	Age and gender

* The results were derived from the semi-structured interviews.

Considering that more than seven attributes in a DCE would be too burdensome for respondents (37), we identified seven attributes based on (1) their comprehensiveness and easiness to measure (e.g., “patient request for antibiotics” was selected, but not “perceived value of antibiotics” for measuring “antibiotic literacy”); (2) the existing evidence in the literature regarding their impacts on antibiotic prescribing decision making (e.g., patient-physician relationship and service arrangements); (3) the clinical justification for antibiotic prescribing (e.g., patient age and duration of symptoms); and (4) their coverage of all the themes. The inclusion of clinical justification served the purpose of minimizing bias (38), which also allowed us to estimate the relative importance of different attributes. In this study, the patient-physician relationship was measured by interpersonal familiarity. Out-of-pocket payment for medicines represented a consideration of affordability of patients. Follow-up appointment and prescribing filling measured service arrangements (Table 2).

**Table 2:** Design of patient attributes and corresponding levels for the discrete choice experiment.

**Attributes**	**Level 1**	**Level 2**	**Level 3**
Age (years)	18–59	60–75	>75
Duration of symptoms (days)	3	6	9
Follow-up appointment	Difficult to schedule	Easy to schedule	
Familiarity	Stranger	Acquaintance	Relative/friend
Request for antibiotics	Indicating not wanting antibiotics unless necessary	No expression of want	Indicating wanting antibiotics
Out-of-pocket payment for medicines	Indicating a maximal out of pocket payment of 30 CNY	Willing to pay for all medicines out of pocket	Expense of medicines partly reimbursed by health insurance
Prescription filling	Outside of the primary care facility	Within the primary care facility	

The levels of each attribute measured in this study were determined in line with the literature and the interview data, based on the assumption that they would make a difference in prescribing decision making. To simplify the DCE, the attribute of “age” was restricted to adults (≥18 years). According to the WHO, 60–74 years of age is deemed young elderly, while 75–89 years of age is deemed old elderly (39). The duration of symptoms was graded around the mean duration (7–10 days) of common colds (40). The levels for follow-up appointment and request for antibiotics followed the DCE design of Lum et al. (27) (Table 2).

#### 2.3.2. Experiment design

Forced choice between two choice profiles was employed in the DCE design. Opt-out options were not included because of concerns that respondents would avoid challenging choices, which might result in insufficient data (41). Effects coding was used because of the largely qualitative nature of the attribute levels. The main effects were estimated through optimal orthogonal in the differences design which assumes zero priors. To reduce the response burden, we assigned all the choice sets into three blocks, each containing 12 choice sets in line with the recommendations in the literature (38). The syntax created for input into Ngene software version 1.2 with three blocks generated 36 rows and a desirable D-optimality of 100%, including efficiency and a correction matrix of zero (C matrix).

Each participant was assigned randomly to one of these three blocks and completed the choice tasks through a questionnaire survey administered in face-to-face interviews. The questionnaire comprised three sections. The first section described a clinical case of a predefined patient with a URTI:

“*An adult patient with a runny nose, sneezing, sore throat, and dry cough visits for your help. There is no specific medical history. Upon examination, the patient has a body temperature of 37.8*°*C and a slight redness of the throat with no exudate. Cervical lymph nodes are normal without enlargement upon observation. No aberration is found from chest X-ray and blood tests. The patient claims to have self-administered OTC medicines for cold and cough prior to the visit, but the symptoms persist*.”

**Figure 1:**
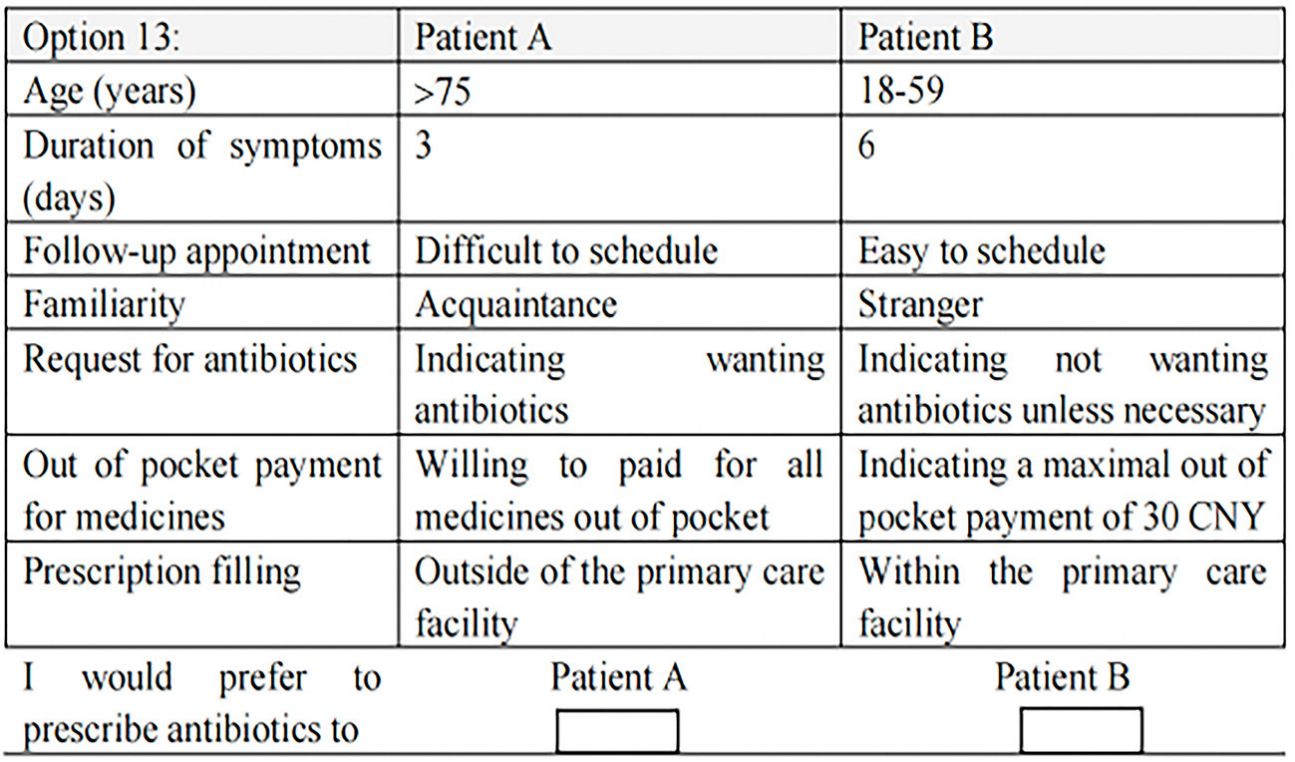
An example of choice sets for the discrete choice experiment.

The second section asked the respondents to make a forced choice with the 12 choice sets. One choice set was also duplicated to verify the reliability of the responses. Figure 1 provides an example of a choice set.

The third section asked for the sociodemographic characteristics of the respondents, including age, sex, professional title, and highest qualification.

A pilot test of the questionnaire was conducted on 15 primary care physicians conveniently selected in Wuhan, the capital city of Hubei province. They were asked to review the case scenario and complete the DCE choice tasks. Feedback on the clarity of the task instructions, the relevance of the choice sets to their practice, and potential misunderstandings was collected. This resulted in some minor modifications to the wording, such as those describing the symptoms in the first section.

#### 2.3.3. Study participants and data collection

The DCE was conducted over the period from August to September 2019. A stratified cluster sampling strategy was adopted to select the study participants. The 17 municipalities in Hubei province were first ranked in order using a comprehensive TOPSIS (Technique for Order Preference by Similarity to Ideal Solution) score containing the following 10 indicators, per capita GDP, population size, per capita disposable income of urban residents, per capita disposable income of rural residents, hospital beds per 1,000 inhabitants, doctors per 1,000 inhabitants, nurses per 1,000 inhabitants, total retail sales of consumer goods, local public finance revenue, and total export-import volume (Appendix 2). One municipality from the top (Wuhan), middle (Jingmen), and bottom (Qianjiang) range was selected randomly. The disposable income of residents in Wuhan (42,133 CNY) was higher than the national average (28,228 CNY), while Jingmen (26,073 CNY) and Qianjiang (24,523 CNY) were lower than the national average. The study setting was further narrowed to Huangpi district in Wuhan and Shayang county in Jingmen due to their large number of primary care institutions. Huangpi's GDP ranked in the middle (seventh) of the 14 districts/counties in Wuhan in 2018 (42), while Shayang's GDP ranked fourth in the five districts/counties in Jingmen (43).

A total of 29 primary care institutions (11 in Huangpi, 10 in Qianjiang, nine in Shayang) agreed to participate in this study. All of the primary care physicians with a right to prescribe antibiotics from these participating institutions were invited to participate in the DCE. This resulted in a final sample of 398 primary care physicians (148 from Huangpi; 150 from Qianjiang, and 100 from Shayang), representing 96.1% of the primary care physicians employed by the participating institutions. The sample size was more than five times the minimal requirement, according to Orme's rule of thumb formula (44).

Data were collected through a questionnaire survey administered in face-to-face interviews. A group of investigators with a bachelor's degree or higher were trained prior to the field work to ensure that all of the protocols had been followed properly. Data entry was conducted by two researchers (XT and LZ) using Microsoft Excel version 2019 to ensure accuracy.

### 2.4. Data analysis

The DCE data comprised 12 stated choices per participant, each indicating a preference between two varying scenarios. The statistical analysis followed the guidelines from the ISPOR (45), using STATA version 15 (StataCorp 2018).

The conditional logit (CL) model and mixed logit (MXL) model were established in line with the random utility theory (46). The utility function is specified as follows:


(1) $$\begin{array}{l} \begin{array}{c} U_{ijr}=\beta_{i}X_{ijr}+E_{ijr} \end{array} \end{array}$$

Where *U*_*ijr*_ is the utility individual *i* derived from scenario *j* in the choice set *r* (here *j* = 1, 2; *r* = 1, …, 12); *X*_*ijr*_ is a vector of the observed attribute (i.e., certain age of patients); β_*i*_ is a vector of coefficient reflecting the desirability of the attribute, which indicates the effects of the predictor on the logarithm of the odds of being in one category vs. the reference category, where the odds in this study represent the ratio of the probability of participant *i* prescribing antibiotics to the probability of not prescribing antibiotics; ε_*ijr*_ is an error term that captures the influence of unobserved factors.

We started with a fitted CL model, which describes the general preference pattern of the respondents. The preferences of all respondents were assumed to be identical, indicating no individual variations in the coefficient.

We then established an MXL model with correlated normally distributed random coefficients to estimate the average preference that allows preference heterogeneity across respondents. In the MXL model, individual preferences are assumed to have a multivariate normal distribution in the population (47), and a full covariance matrix among the randomly distributed utility coefficients can be estimated (48). The utility for individual *i* associated with scenario *j* in the choice set *r* is:


(2) $$\begin{array}{l} \begin{array}{c} U_{ijr}=\beta_{i}^{{}^{\prime }}X_{ijr}+\varepsilon_{ijr}=\bar{\beta }X_{ijr}+\tau_{i}X_{ijr}+\varepsilon_{ijr} \end{array} \end{array}$$

Where $\bar{\beta }$ is the mean preference vector for the population, and τ_*i*_ is a multivariate normal distributed vector.

The attributes with a random distribution of parameters were identified using t-statistics for standard deviations (49). The likelihood-ratio test (50) showed that none of the standard deviations of the attribute parameters are equal to zero (*p* < 0.001). The t-statistics indicated significant preference heterogeneity for the following attributes (Appendix 3): >75 years (*z* = 11.2, *p* < 0.001), 6 days of symptom duration (*z* = 2.4, *p* < 0.05), 9 days of symptom duration (*z* = 11.4, *p* < 0.001), and an indication of wanting antibiotics (*z* = 5.7, *p* < 0.001). Thus, both the fixed and random effects of these attributes were estimated in the MXL. The coefficients of random parameters were presupposed to follow a normal distribution (51). The MXL model was iterated with 500 Halton draws.

The relative importance (RI) of each attribute was calculated by dividing the utility range of each attribute with the utility range total (52). We also tested the interaction between the clinical attributes (i.e., age and duration of symptom) and the interventionable patient attributes in the MXL model (53).

The performance of the CL and MXL models was compared using logarithmic likelihood (LL), Akaike information criterion (AIC), and Bayesian information criterion (BIC). Higher LL, lower AIC, and lower BIC indicate higher performance (45).

## 3. Results

Of the 398 study participants, 12 failed to provide a consistent choice on the duplicated choice tasks. This resulted in a final sample size of 386 respondents, containing 9,166 choice data points. The respondents had a mean age of 42.17 years (SD = 9.7) and had worked, on average, 19.4 years (SD = 10.7) in the health sector. Most (72.3%) were male physicians, which is higher than the national average (59.4%). About half had obtained an associate medical degree and had a junior professional title. The vast majority (98.4%) worked in rural township health centers, received educational materials about antibiotic prescribing (95.1%), and attended relevant training over the past year (84.2%). The study sample resembled the characteristics of the workforce of primary care (assistant) physicians in rural township health centers in China in 2018 (Table 3).

**Table 3:** Demographic characteristics of study participants in discrete choice experiment.

**Characteristics**	**Mean ± SD or *n* (%)**	China^*^(%)
**Age (years)**	42.17 ± 9.66	-
< 25	8 (2.07)	0.30
25–34	72 (18.65)	16.50
35–44	149 (38.60)	37.50
45–54	118 (30.57)	32.50
55–59	26 (6.74)	6.70
≥60	13 (3.37)	6.50
Male gender	279 (72.28)	59.40
**Work experience (years)**	19.39 ± 10.66	-
< 5	39 (10.10)	7.90
5–9	38 (9.84)	14.60
10–19	87 (22.54)	23.10
20–29	151 (39.12)	35.70
≥30	71 (18.39)	18.80
**Professional title**		
Senior title	4 (1.03)	0.32
Vice-senior title	20 (5.18)	4.75
Middle title	142 (36.79)	21.96
Primary title	220 (57.00)	72.97
**Highest qualification**		
Vocational training	51 (17.83)	35.30
Associate degree	188 (48.71)	43.80
University degree	147 (38.08)	20.90
**Annual household income (CNY)**		
< 40,000	105 (27.20)	-
40,000–59,999	142 (36.79)	-
60,000–79,999	67 (17.36)	-
80,000–99,999	42 (10.88)	-
≥100,000	30 (7.77)	-
**Department**		
Internal medicine	115 (29.79)	-
Surgical	46 (11.92)	-
General practice	84 (21.76)	-
Others	141 (36.53)	-
**Institution**		
Urban community health center	5 (1.30)	-
Rural township health center	381 (98.70)	-
Receiving educational materials on antibiotic prescribing	367 (95.08)	-
Attending antibiotic training course over the past year	329 (84.23)	-

SD, standard deviation; CNY, Chinese Yuan.

* Data extracted from the 2019 edition of China Health Statistical Yearbook; - Data not available.

### 3.1. Conditional logit and mixed logit models

The CL model showed that the respondents preferred to prescribe antibiotics for patients who were older [relative to < 60 years, β = 0.39 (0.29, 0.48) for 60–75 years; β = 0.45 (0.35, 0.55) for >75 years], had experienced a prolonged duration of symptoms [relative to 3 days, β = 0.86 (0.77, 0.96) for 6 days; β = 1.6 (1.5, 1.7) for 9 days], and felt it was difficult to schedule a follow-up appointment [relative to difficult to schedule a follow-up appointment, β = −0.15 (−0.21, −0.08) for easy to schedule a follow-up appointment]. The patients who clearly expressed a refusal for antibiotics unless necessary were less likely to be prescribed antibiotics than those who had made no such expressions [β = 0.27 (0.17, 0.37)] or those who had positively requested antibiotics [β = 0.42 (0.32, 0.51)]. Familiarity with prescribers, out-of-pocket payments for medicines, and choice of settings to fill prescriptions played no significant role in antibiotic prescribing decisions.

The MXL model yielded similar results: older age, prolonged duration of symptoms, difficulties in scheduling a follow-up appointment, and absence of a specific expression of refusal for unnecessary antibiotics were significant predictors of antibiotic prescribing preference (Table 4). The MXL model performed substantially better than the CL model as indicated by its higher LL (−2,378.8 vs. −2,521.8), lower AIC (4,789.5 vs. 5,067.5), and lower BIC (4,903.5 vs. 5,153.0).

**Table 4:** Results of conditional logit model and mixed logit model against the data set of the discrete choice experiment.

**Attributes**	**Levels**	**Conditional logit (CL) model**				**Mixed logit (MXL) model**			
		**β**	**Standard error**	***P***	**95% confidence interval**	**β**	**Standard error**	***P***	**95% confidence interval**
**Age (base** = **younger than 60 years)**									
60–75 years		0.39	0.05	< 0.001	[0.29, 0.48]	0.35	0.07	< 0.001	[0.22, 0.48]
>75 years		0.45	0.05	< 0.001	[0.35, 0.55]	0.52	0.08	< 0.001	[0.37, 0.68]
Random effect (>75 years)						0.97	0.08	< 0.001	[0.81, 1.13]
**Duration of symptoms (base** = **3 days)**									
6 days		0.86	0.05	< 0.001	[0.77, 0.96]	1.02	0.06	< 0.001	[0.90, 1.13]
Random effect (6 days)						0.32	0.14	0.03	[0.04, 0.60]
9 days		1.62	0.05	< 0.001	[1.52, 1.73]	2.23	0.11	< 0.001	[2.00, 2.45]
Random effect (9 days)						1.39	0.12	< 0.001	[1.17, 1.62]
**Follow-up appointment (base** = **difficult to schedule)**									
Easy to schedule		−0.15	0.04	< 0.001	[−0.21, −0.08]	−0.19	0.04	< 0.001	[−0.28, −0.11]
**Familiarity (base** = **stranger)**									
Acquaintance		−0.02	0.05	0.68	[−0.12, 0.08]	−0.07	0.06	0.22	[−0.19, 0.04]
Relative/friend		−0.02	0.05	0.66	[−0.12, 0.08]	−0.02	0.06	0.77	[−0.14, 0.10]
**Request for antibiotics (base** = **indicating not wanting antibiotics unless necessary)**									
No expression of want		0.27	0.05	< 0.001	[0.17, 0.37]	0.32	0.06	< 0.001	[0.21, 0.44]
Indicating wanting antibiotics		0.42	0.05	< 0.001	[0.32, 0.51]	0.60	0.07	< 0.001	[0.46, 0.73]
Random effect (indicating wanting antibiotics)						0.52	0.09	< 0.001	[0.34, 0.71]
**Out of pocket payment for medicines (base** = **indicating a maximal out of pocket payment of 30 CNY)**									
Willing to paid for all medicines out of pocket		0.08	0.05	0.12	[−0.02, 0.17]	0.05	0.06	0.45	[−0.07, 0.17]
Expense of medicines partly reimbursed by health insurance		0.00	0.05	0.98	[−0.10, 0.10]	−0.03	0.06	0.64	[−0.14, 0.09]
**Prescription filling (base** = **outside of the primary care facility)**									
Within the primary care facility		0.02	0.04	0.62	[−0.05, 0.09]	0.04	0.04	0.33	[−0.04, 0.12]
Number of respondents		386				386			
Number of observations		9,166				9,166			
Log likelihood		−2,521.76				−2,378.76			
Akaike information criterion		5,067.52				4,789.51			
Bayesian information criterion		5,153.00				4,903.49			

### 3.2. Relative importance of attributes

**Figure 2:**
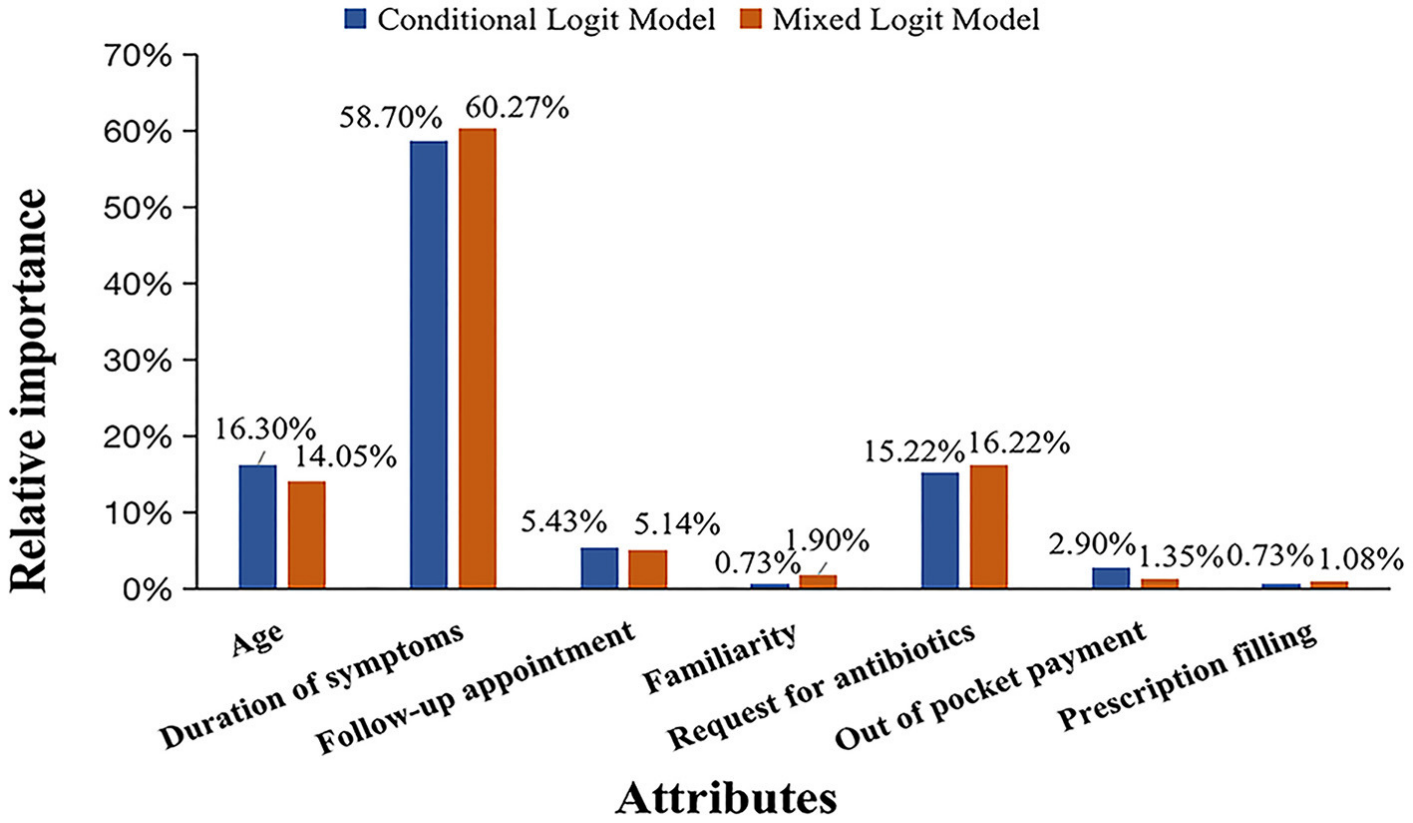
Relative importance of the tested attributes in conditional logit and mixed logit models.

Duration of symptoms was identified as the most important attribute that influenced the preference of antibiotic prescribing in both the CL model (RI = 58.7%) and the MXL model (RI = 60.3%). The influence of patient age and request for antibiotics came almost equally second, with RI ranging from 14.1 to 16.3% in the two models. Easiness of follow-up appointment had 5.1 and 5.4% RI in the CL model and MXL model respectively, whereas, the rest of the attributes had lower than 5.0% RI (Figure 2).

The interaction testing showed that the influence of the interventionable patient attributes was most profound in the antibiotic prescribing decisions for patients aged between 60 and 75 years as indicated by their interaction effects with age (β = 0.69 for request for antibiotics, *p* < 0.01; β = −1.2 for easiness of follow-up, *p* < 0.001; Table 5).

**Table 5:** Interaction effects between clinical and non-clinical factors: results of mixed logit model.

**Interaction terms**	**β**	**Standard error**	***P***	**95% confidence interval**
60–75 years^*^ Easy to schedule follow-up appointment	**−1.22**	**0.37**	**< 0.001**	**[−1.95**, **−0.50]**
>75 years^*^ Easy to schedule follow-up appointment	−0.05	0.34	0.89	[−0.72, 0.63]
6 days^*^ No expression of want	0.05	0.26	0.85	[−0.47, 0.56]
60–75 years^*^ Indicating wanting antibiotics	**0.69**	**0.23**	**< 0.01**	**[0.25, 1.14]**
>75 years^*^ Indicating wanting antibiotics	0.32	0.21	0.13	[−0.10, 0.73]
9 days^*^ Indicating wanting antibiotics	0.29	0.25	0.25	[−0.20, 0.78]

Bold indicates statistical significance at the 0.05 level. The * symbol indicates the multiplication.

The preference heterogeneity analyses showed that the older and more experienced physicians were less likely to prescribe antibiotics to patients who requested them as indicated by its interaction effects with age [β = −0.02 (−0.03, −0.01)] and work experience [β = −0.02 (−0.03, −0.01); Appendix 4].

## 4. Discussion

The findings of our study show that clinical justification (e.g., age and duration of symptoms) remains the most important consideration of primary care physicians in Hubei of China in prescribing antibiotics for URTIs. However, patient request for antibiotics and concerns about the accessibility and continuity of care also have a significant impact on antibiotic prescribing decisions, albeit with a lower relevant importance.

Patient request for antibiotics can drive antibiotic prescribing for URTIs. We found that not only patient request for antibiotics but also the absence of a clear indication of refusal for antibiotics can trigger a higher willingness of primary care physicians to prescribe antibiotics for URTIs. This is concerning. Physicians often overestimate patient expectations when patients do not make a specific request (54) and may assume that these patients follow popular public opinion. In China, antibiotics are often tagged as “anti-inflammatory,” leading to a belief in the inflated effects of antibiotic treatment (55). Internationally, primary care physicians are usually not well-prepared to manage high patient expectations on antibiotics due to time constraints, diagnostic uncertainty, and poor communication skills (56).

Difficulties in scheduling follow-up appointments present a barrier for containing antibiotic prescriptions for URTIs in primary care. We found that primary care physicians may defer potential antibiotic prescribing for URTIs if a follow-up appointment can be easily scheduled. Internationally, the strategy of delayed antibiotic prescriptions is often used in response to patient demands for antibiotic prescriptions (57). However, such a strategy can encounter several barriers in China. Firstly, access to health facilities in some remote rural communities is often poor, despite unprecedented socioeconomic development over the past few decades in China (58). Prescribing antibiotics may help patients reduce the need for a return visit to health facilities (59). Secondly, the effect of delayed prescriptions on reducing antibiotic consumption can be compromised by poor patient compliance, that is, patients do not decide whether to take antibiotics based on their own disease outcomes as required by their physicians, but take them immediately (60). Finally, patients in China may be able to purchase antibiotics from commercial pharmacy retail outlets (a common problem in many low- and middle-income countries). This makes primary care physicians feel less hesitant to prescribe antibiotics (61).

We found that familiarity with prescribers, out-of-pocket payments for medicines, and choice of settings to fill prescriptions play no significant roles in the antibiotic prescribing decisions of primary care physicians. These results are not always consistent with the findings of previous studies. In a qualitative study in Europe, the researchers found that in the case of lower respiratory tract infections, the interviewed primary physicians tended to prescribe antibiotics earlier to patients they knew well (62). For unfamiliar patients, Poss-Doering et al. in a study of 27 primary care physicians in Germany found that the participants acknowledged inappropriate antibiotic prescribing for acute, non-complicated, and self-limiting infections (57).

Out-of-pocket payment requirements are an important consideration in prescribing decisions when consumer affordability is a major concern. However, primary care institutions in China are only allowed to prescribe medicines listed on the essential medicines list, which are usually linked to low prices (63). This may explain why financial burden is not considered by primary care physicians in their prescribing preference.

In China, patients used to be encouraged to fill prescriptions within the health facilities in which the prescribers were employed for financial gains. However, primary care institutions have no longer been able to make any financial profit from the sales of medicines since the zero-markup policy was introduced in 2009. This policy has substantially lowered the prices of medicines and reduced antibiotic prescriptions (64). This may explain why patient choice of settings to fill prescriptions plays no significant role in the antibiotic prescribing decisions of primary care physicians.

China has made great progress in curbing the over-prescription of antibiotics (65). However, some new challenges emerged after decades of efforts resulting from several rounds of health reforms. The major driving force underlying the over-prescription of antibiotics has shifted from perverse financial incentives for providers to pressures from consumers. While consumer demands and expectations of healthcare services are increasing, the balance of power between patients and providers is changing. Health workers are becoming increasingly concerned about the deteriorating patient-provider relationship (66). Internationally, several tools have been tested in helping primary care physicians to reduce antibiotic prescriptions, such as using C-reactive protein (CRP) to test bacterial infections (67), providing social norm feedback and targeted education for high antibiotic prescribers (54), educating URTI patients about antibiotic use in addition to improving the communication skills of primary care physicians (56, 68–70). According to our findings by preference heterogeneity analyses, younger and less experienced primary care physicians should be prioritized for behavioral intervention training, especially in relation to managing older patients. More empirical study on the impact of patient symptom duration and their age on physician antibiotic prescribing behavior is needed in the future. Further action in reducing the over-use of antibiotics must include strategies relating to the introduction of more detailed guidelines for primary clinical diagnosis and treatment of URTIs, including different symptoms and corresponding symptom duration.

There are several limitations in this study. Firstly, the forced nature of choice in DCEs limits this study's ability to reflect on realistic decisions. The DCE design included seven patient attributes with 12 choice tasks. This may present a high cognitive burden for some study participants. Although there is no golden rule about the optimal number of attributes in DCEs (71), a high number of attributes decreases the reliability of the results (72). Although the sample size of our study is quite large for a DCE design, the study participants were drawn from primary care institutions in Hubei province. Attempts to generalize the results to the entire country and other health sectors need to be made cautiously.

## 5. Conclusion

Although clinical justification has always been the paramount consideration in antibiotic prescribing decisions, primary care physicians in China are under significant pressure from patient requests for antibiotics which may jeopardize clinical appropriateness when making prescription decisions. The lack of seamless care arrangements in the healthcare delivery system may also diminish patients' access, especially those from remote rural areas, to primary health care, resulting in primary physicians' aggressive antibiotic prescribing behaviors. Reducing patient pressure and improving accessibility and continuity of care may help primary care physicians make rational antibiotic prescribing decisions for URTIs.

## Data availability statement

The raw data supporting the conclusions of this article will be made available by the authors, without undue reservation.

## Ethics statement

This study was approved by the Research Ethics Committee of Tongji Medical College, Huazhong University of Science and Technology (No. IORG0003571). Oral informed consent was obtained from each participant prior to data collection. The patients/participants provided their written informed consent to participate in this study.

## Author contributions

TX conducted the literature review, designed the analysis framework, contributed to the data cleaning, preformed formal analysis, and wrote the original draft. YT designed the study, obtained funding, contributed to the interpretation of the results, and preformed revisions of the manuscript. CL contributed to the interpretation of the results and preformed revisions of the manuscript. ZL and JL took part in the investigation, contributed to the qualitative research, and performed revisions of the manuscript. All authors contributed to the article and approved the submitted version.
