# Supplementary material for: Weighing patient attributes in antibiotic prescribing for upper respiratory tract infections: A discrete choice experiment on primary care physicians in Hubei Province, China
Source: Front Public Health. 2022 Dec 20;10:1008217. doi: 10.3389/fpubh.2022.1008217 (PMC9807867; doi:10.3389/fpubh.2022.1008217)
Supplement: Supplementary file 1 [file Data_Sheet_1.PDF]

Appendix 1 - Demographic characteristics of participants in semi-structured interviews

Table 1. Demographic characteristics of 13 participants in semi-structured interviews

**Variables**	**Mean ± SD or n (%)**
Age	49.23 ± 11.92
Work experience (Years)	27.85 ± 13.29
Gender (Male)	12 (92.31)
Educational attainment	
≤ High school	2 (15.39)
Associate degree	9 (69.22)
≥ Bachelor's degree	2 (15.39)
Professional title	
Physician assistant	3 (23.08)
Resident physician	7 (53.84)
Attending physician	3 (23.08)
Department	
Internal medicine	6 (46.15)
Traditional Chinese medicine	1 (7.69)
Integrated traditional and western medicine	2 (15.39)
General practice	4 (30.77)

SD - standard deviation

Appendix 2 – TOPSIS results

Table 2. The results of TOPSIS score of 17 municipalities in Hubei province, China

Municipality	Per capita GDP (CNY)	Population size (10,000)	Urban per capita disposable income (CNY)	Rural per capita disposable income (CNY)	Hospital beds per thousand population	Number of doctors per thousand population	Number of nurses per thousand population	Total retail sales of consumer goods (100 million CNY)	Local public revenue (100 million CNY)	Total export-import (100 million CNY)	Homogenize score
Wuhan	135877	1108.10	47359	22652	8.60	3.57	4.91	6843.90	1528.70	2146.00	0.2851
Xiangyang	76024	566.9	33947	17305	6.71	2.48	2.80	1658.96	295.52	188.40	0.0734
Yichang	98269	413.59	35011	16514	7.07	2.73	3.58	1484.01	237.24	202.20	0.0705
Huanggang	25010	633.00	28978	13238	5.89	2.02	2.26	1205.05	139.24	61.90	0.0572
Jingzhou	37076	559.02	32590	17300	5.61	2.24	2.50	1298.65	134.31	120.00	0.0569
Shiyan	54714	340.60	30771	10295	8.73	3.04	3.84	915.02	113.30	45.90	0.0507
Xiaogan	38900	492.00	32685	15988	5.04	1.88	2.28	1085.47	130.19	92.40	0.0496
Huangshi	65206	247.07	35327	15125	6.66	2.42	3.81	803.27	117.02	248.50	0.0482
Jingmen	63742	289.65	33779	18776	6.12	2.42	3.03	772.51	105.76	108.70	0.0449
Ezhou	93317	107.77	31742	17609	5.62	2.06	2.84	379.04	57.93	43.20	0.042
Xianning	57270	254.33	30337	15116	5.76	2.72	3.07	556.18	91.32	36.70	0.0371
Xiantao	70156	114.00	31672	18177	4.92	2.40	3.52	373.15	33.55	67.30	0.037
Enshi	25848	337.80	28918	10524	7.20	2.40	3.24	616.83	80.21	5.70	0.0369
Qianjiang	78279	96.60	31574	17797	4.72	2.30	2.72	260.11	25.84	46.30	0.0353
Suizhou	45681	221.67	29237	16538	5.16	1.83	2.08	545.58	47.38	65.20	0.0286
Shennongjia	36843	7.76	28176	10091	6.70	2.96	3.17	18.44	5.10	0.00	0.0239
Tianmen	46259	127.23	28825	16598	5.04	2.00	2.27	359.55	20.40	8.90	0.0226

CNY - Chinese Yuan

Appendix 3 – Results of the t-test for standard deviation

Table 3. Result of the specification test (t-test for standard deviation)

Attributes	Levels	*β*	Standard	*z*-score	*p*-value	95 % Confidence
Mean						
Age (Base = Younger than 60 years)						
	60-75 years	0.36	0.07	5.24	< 0.001	[0.23,0.50]
	> 75 years	0.54	0.08	6.57	< 0.001	[0.38,0.70]
Duration of symptoms (Base = 3 days)						
	6 days	1.05	0.07	15.88	< 0.001	[0.92,1.18]
	9 days	2.31	0.13	17.84	< 0.001	[2.06,2.57]
Follow-up appointment (Base = Difficult to schedule)						
	Easy to schedule	-0.21	0.05	-4.53	< 0.001	[-0.30, -0.12]
Familiarity (Base = Stranger)						
	Acquaintance	-0.08	0.06	-1.24	0.22	[-0.20,0.05]
	Relative/friend	-0.02	0.06	-0.24	0.81	[-0.14,0.11]
Desire for antibiotics (Base = Indicating not wanting antibiotics unless necessary)						
	No expression of want	0.34	0.06	5.42	< 0.001	[0.21,0.46]
	Indicating wanting antibiotics	0.62	0.07	8.60	< 0.001	[0.48,0.76]
Out of pocket payment for medicines (Base = Indicating a maximal out of pocket payment of 30 CNY)						
	Willing to paid for all medicines out of pocket	0.05	0.06	0.77	0.44	[-0.08,0.17]
	Expense of medicines partly reimbursed by health insurance	-0.03	0.06	-0.46	0.65	[-0.15,0.09]
Prescription filling (Base = Outside of the primary care facility)						
	Within the primary care facility	0.04	0.05	0.92	0.36	[-0.05,0.13]
Standard Deviation						

Age (Base = Younger than 60 years)					
60-75 years	0.02	0.14	0.12	0.90	[-0.25,0.29]
> 75 years	1.02	0.09	11.17	< 0.001	[0.84,1.20]
Duration of symptoms (Base = 3 days)					
6 days	0.34	0.14	2.38	0.02	[0.06,0.62]
9 days	1.46	0.13	11.42	< 0.001	[1.21,1.71]
Follow-up appointment (Base = Difficult to schedule)					
Easy to schedule	0.17	0.14	1.23	0.22	[-0.10,0.43]
Familiarity (Base = Stranger)					
Acquaintance	0.09	0.22	0.38	0.70	[-0.35,0.52]
Relative/friend	0.10	0.16	0.63	0.53	[-0.21,0.40]
Desire for antibiotics (Base = Indicating not wanting antibiotics unless necessary)					
No expression of want	0.00	0.17	0.01	0.99	[-0.33,0.34]
Indicating wanting antibiotics	0.55	0.10	5.70	< 0.001	[0.36,0.74]
Out of pocket payment for medicines (Base = Indicating a maximal out of pocket payment of 30 CNY)					
Willing to paid for all medicines out of pocket	0.18	0.19	0.95	0.35	[-0.19,0.54]
Expense of medicines partly reimbursed by health insurance	0.09	0.14	0.63	0.53	[-0.19,0.37]
Prescription filling (Base = Outside of the primary care facility)					
Within the primary care facility	0.35	0.09	4.09	< 0.001	[0.18,0.51]
Model diagnostics					
Number of respondents			386		
Number of observations			9166		
Log Likelihood			-2376.74		
Akaike information criterion			4801.48		
Bayesian information criterion			4972.44		

CNY - Chinese Yuan

Appendix 4 – Results of the interaction effect model

Preference heterogeneity analysis was conducted with the interaction terms of physicians' age, gender, work experience, institution, department, professional title, annual household income, educational attainment, whether they received educational materials on antibiotic prescribing and whether they attended antibiotic training course over the past year. The reference groups for gender, institution, department, professional title, annual household income, educational attainment, whether receiving educational materials on antibiotic prescribing and whether attending antibiotic training course over the past year were female, urban community health centres, internal medicine, primary title, <40,000 CNY, vocational training, having not received educational materials on antibiotic prescribing and having not attended antibiotic training course over the past year, respectively.

The results shown in Table 4 and Table 6 indicate that older physicians and more experienced physicians were more reluctant to prescribe antibiotics to patients with prolonged symptoms and who required antibiotics. As shown in Table 7, physicians in rural township health centres prefer to prescribe antibiotics to patients over 75 and those with symptoms lasting 9 days compared to physicians in urban community health centres. It can be seen in Table 8 that compared with internal medicines, surgeons and physicians in other departments were reluctant to prescribe antibiotics to patients with symptoms lasting 9 days. The results in Table 10 and Table 11 show that physicians with annual household incomes of 40,000- 59,999 CNY (relative to <40,000 CNY) and those with a university degree (relative to vocational training) were more likely to prescribe antibiotics to patients with longer duration of symptoms. There were no statistically significant attribute interactions with gender, professional title, whether receiving educational materials on antibiotic prescribing and whether attending antibiotic training course over the past year are not statistically significant, indicating no preference heterogeneity among these four demographic characteristics.

Table 4. Interaction effects model estimation for participated physicians' age

Attributes	Levels	Interaction effect model 1 - Age			
		β	**Standard Error**	***p***	**95 % Confidence Interval**
Age (Base = Younger than 60 years)					
	60-75 years	0.35	0.07	< 0.001	[0.22, 0.48]
	> 75 years	1.07	0.32	< 0.001	[0.44, 1.69]
	Random effect (> 75 years)	0.97	0.08	< 0.001	[0.80, 1.13]
Duration of symptoms (Base = 3 days)					
	6 days	1.68	0.26	< 0.001	[1.17, 2.18]
	Random effect (6 days)	0.31	0.15	0.03	[0.03, 0.60]
	9 days	3.27	0.44	< 0.001	[2.41, 4.14]
	Random effect (9 days)	1.39	0.12	< 0.001	[1.16, 1.61]
Follow-up appointment (Base = Difficult to schedule)					

	Easy to schedule	-0.19	0.04	< 0.001	[-0.27, -0.11]
Familiarity (Base = Stranger)					
	Acquaintance	-0.08	0.06	0.18	[-0.20, 0.04]
	Relative/friend	-0.02	0.06	0.71	[-0.14, 0.10]
Desire for antibiotics (Base = Indicating not wanting antibiotics unless necessary)					
	No expression of want	0.32	0.06	< 0.001	[0.21, 0.44]
	Indicating wanting antibiotics	1.22	0.26	< 0.001	[0.64, 1.35]
	Random effect (Indicating wanting antibiotics)	0.52	0.09	< 0.001	[0.71, 1.73]
Out of pocket payment for medicines (Base = Indicating a maximal out of pocket payment of 30 CNY)					
	Willing to paid for all medicines out of pocket	0.05	0.06	0.44	[-0.07, 0.17]
	Expense of medicines partly reimbursed by health insurance	-0.03	0.06	0.64	[-0.15, 0.09]
Prescription filling (Base = Outside of the primary care facility)					
	Within the primary care facility	0.04	0.04	0.32	[-0.04, 0.12]
Interaction terms					
> 75 years*Age (physicians)		-0.01	0.01	0.08	[-0.03, 0.00]
6 days *Age (physicians)		**-0.02**	**0.01**	**0.01**	**[-0.03, -0.01]**
9 days *Age (physicians)		**-0.03**	**0.01**	**0.01**	**[-0.04, -0.01]**
Indicating wanting antibiotics *Age (physicians)		**-0.02**	**0.01**	**0.01**	**[-0.03, -0.01]**
Model diagnostics					
	Number of respondents		386		
	Number of observations		9166		
	Log Likelihood		-2369.97		
	Akaike information criterion		4779.95		
	Bayesian information criterion		4922.41		

CNY - Chinese Yuan; Bold font indicates statistical significance at the 0.05 level

Table 5. Interaction effects model estimation for participated physicians' gender

Attributes	Levels	Interaction effect model 2 - Gender			
		β	**Standard Error**	***p***	**95 % Confidence Interval**
Age (Base = Younger than 60 years)					
	60-75 years	0.35	0.07	< 0.001	[0.22, 0.48]
	> 75 years	0.54	0.14	< 0.001	[0.26, 0.81]
	Random effect (> 75 years)	0.97	0.08	< 0.001	[0.81, 1.14]
Duration of symptoms (Base = 3 days)					
	6 days	1.11	0.11	< 0.001	[0.89, 1.33]
	Random effect (6 days)	0.31	0.14	0.03	[0.03, 0.60]
	9 days	2.22	0.19	< 0.001	[1.85, 2.60]

	Random effect (9 days)	1.39	0.12	< 0.001	[1.16, 1.62]
Follow-up appointment (Base = Difficult to schedule)					
	Easy to schedule	-0.19	0.04	< 0.001	[-0.28, -0.11]
Familiarity (Base = Stranger)					
	Acquaintance	-0.08	0.06	0.21	[-0.19, 0.04]
	Relative/friend	-0.02	0.06	0.77	[-0.14, 0.10]
Desire for antibiotics (Base = Indicating not wanting antibiotics unless necessary)					
	No expression of want	0.32	0.06	< 0.001	[0.21, 0.44]
	Indicating wanting antibiotics	0.60	0.11	< 0.001	[0.37, 0.82]
	Random effect (Indicating wanting antibiotics)	0.53	0.09	< 0.001	[0.34, 0.71]
Out of pocket payment for medicines (Base = Indicating a maximal out of pocket payment of 30 CNY)					
	Willing to paid for all medicines out of pocket	0.05	0.06	0.46	[-0.07, 0.17]
	Expense of medicines partly reimbursed by health insurance	-0.03	0.06	0.64	[-0.15, 0.09]
Prescription filling (Base = Outside of the primary care facility)					
	Within the primary care facility	0.04	0.04	0.32	[-0.04, 0.12]
Interaction terms					
> 75 years*Male (physicians)		-0.02	0.16	0.90	[-0.33, 0.29]
6 days * Male (physicians)		-0.13	0.13	0.31	[-0.38, 0.12]
9 days * Male (physicians)		0.00	0.22	0.99	[-0.42, 0.43]
Indicating wanting antibiotics * Male (physicians)		0.00	0.13	0.99	[-0.25, 0.25]
Model diagnostics					
	Number of respondents	386			
	Number of observations	9166			
	Log Likelihood	-2378.17			
	Akaike information criterion	4796.33			
	Bayesian information criterion	4938.80			

CNY - Chinese Yuan; Bold font indicates statistical significance at the 0.05 level

Table 6. Interaction effects model estimation for participated physicians' work experience

Attributes	Levels	Interaction effect model 3 – Work experience			
		β	**Standard Error**	***p***	**95 % Confidence Interval**
Age (Base = Younger than 60 years)					
	60-75 years	0.35	0.07	< 0.001	[0.22, 0.48]
	> 75 years	0.74	0.15	< 0.001	[0.45, 1.04]
	Random effect (> 75 years)	0.97	0.08	< 0.001	[0.81, 1.14]
Duration of symptoms (Base = 3 days)					
	6 days	1.30	0.12	< 0.001	[1.06, 1.54]

	Random effect (6 days)	0.31	0.15	0.03	[0.03, 0.60]
	9 days	2.68	0.23	< 0.001	[2.25, 3.10]
	Random effect (9 days)	1.39	0.12	< 0.001	[1.16, 1.61]
Follow-up appointment (Base = Difficult to schedule)					
	Easy to schedule	-0.19	0.04	< 0.001	[-0.28, -0.11]
Familiarity (Base = Stranger)					
	Acquaintance	-0.08	0.06	0.19	[-0.20, 0.04]
	Relative/friend	-0.02	0.06	0.73	[-0.14, 0.10]
Desire for antibiotics (Base = Indicating not wanting antibiotics unless necessary)					
	No expression of want	0.32	0.06	< 0.001	[0.21, 0.44]
	Indicating wanting antibiotics	0.90	0.13	< 0.001	[0.65, 1.15]
	Random effect (Indicating wanting antibiotics)	0.52	0.09	< 0.001	[0.33, 0.70]
Out of pocket payment for medicines (Base = Indicating a maximal out of pocket payment of 30 CNY)					
	Willing to paid for all medicines out of pocket	0.05	0.06	0.43	[-0.07, 0.17]
	Expense of medicines partly reimbursed by health insurance	-0.03	0.06	0.65	[-0.14, 0.09]
Prescription filling (Base = Outside of the primary care facility)					
	Within the primary care facility	0.04	0.04	0.32	[-0.04, 0.12]
Interaction terms					
> 75 years*Work experience (physicians)		-0.01	0.01	0.09	[-0.02, 0.00]
6 days * Work experience (physicians)		**-0.01**	**0.01**	**0.01**	**[-0.03, -0.01]**
9 days * Work experience (physicians)		**-0.02**	**0.01**	**0.01**	**[-0.04, -0.01]**
Indicating wanting antibiotics * Work experience (physicians)		**-0.02**	**0.01**	**0.01**	**[-0.03, -0.01]**
Model diagnostics					
	Number of respondents		386		
	Number of observations		9166		
	Log Likelihood		-2369.11		
	Akaike information criterion		4778.22		
	Bayesian information criterion		4920.68		

CNY - Chinese Yuan; Bold font indicates statistical significance at the 0.05 level

Table 7. Interaction effects model estimation for participated physicians' institution

Attributes	Levels	Interaction effect model 4 – Institution			
		β	**Standard Error**	***p***	**95 % Confidence Interval**
Age (Base = Younger than 60 years)					
	60-75 years	0.35	0.07	< 0.001	[0.22, 0.48]
	> 75 years	0.68	0.58	0.25	[-1.82, 0.47]
	Random effect (> 75 years)	0.96	0.08	< 0.001	[0.79, 1.12]

Duration of symptoms (Base = 3 days)					
	6 days	0.41	0.48	0.40	[-0.54, 1.35]
	Random effect (6 days)	0.31	0.15	0.04	[0.02, 0.59]
	9 days	0.61	0.78	0.43	[-0.92, 2.15]
	Random effect (9 days)	1.38	0.12	< 0.001	[1.16, 1.61]
Follow-up appointment (Base = Difficult to schedule)					
	Easy to schedule	-0.19	0.04	< 0.001	[-0.27, -0.11]
Familiarity (Base = Stranger)					
	Acquaintance	-0.08	0.06	0.19	[-0.20, 0.04]
	Relative/friend	-0.02	0.06	0.76	[-0.14, 0.10]
Desire for antibiotics (Base = Indicating not wanting antibiotics unless necessary)					
	No expression of want	0.32	0.06	< 0.001	[0.21, 0.44]
	Indicating wanting antibiotics	0.60	0.46	0.19	[-0.30, 1.49]
	Random effect (Indicating wanting antibiotics)	0.52	0.09	< 0.001	[0.33, 0.70]
Out of pocket payment for medicines (Base = Indicating a maximal out of pocket payment of 30 CNY)					
	Willing to paid for all medicines out of pocket	0.05	0.06	0.46	[-0.07, 0.17]
	Expense of medicines partly reimbursed by health insurance	-0.03	0.06	0.67	[-0.14, 0.09]
Prescription filling (Base = Outside of the primary care facility)					
	Within the primary care facility	0.04	0.04	0.32	[-0.04, 0.12]
Interaction terms					
> 75 years*Rural township health centres		**1.22**	**0.59**	**0.04**	**[0.07, 2.37]**
6 days * Rural township health centres		0.61	0.49	0.21	[-0.34, 1.57]
9 days *Rural township health centres		**1.63**	**0.79**	**0.04**	**[0.08, 3.18]**
Indicating wanting antibiotics *Rural township health centres		0.00	0.46	1.00	[-0.90, 0.90]
Model diagnostics					
	Number of respondents	386			
	Number of observations	9166			
	Log Likelihood	-2373.98			
	Akaike information criterion	4787.96			
	Bayesian information criterion	4930.42			

CNY - Chinese Yuan; Bold font indicates statistical significance at the 0.05 level

Table 8. Interaction effects model estimation for participated physicians' department

Attributes	Levels	Interaction effect model 5 – Department			
		β	**Standard Error**	***p***	**95 % Confidence Interval**
Age (Base = Younger than 60 years)					
	60-75 years	0.35	0.07	< 0.001	[0.22, 0.48]

> 75 years	0.56	0.14	< 0.001	[0.29, 0.83]
Random effect (> 75 years)	0.96	0.08	< 0.001	[0.79, 1.12]
Duration of symptoms (Base = 3 days)				
6 days	1.11	0.11	< 0.001	[0.90, 1.32]
Random effect (6 days)	0.30	0.15	0.06	[-0.01, 0.60]
9 days	2.53	0.19	< 0.001	[2.16, 2.90]
Random effect (9 days)	1.35	0.12	< 0.001	[1.12, 1.58]
Follow-up appointment (Base = Difficult to schedule)				
Easy to schedule	-0.19	0.04	< 0.001	[-0.28, -0.11]
Familiarity (Base = Stranger)				
Acquaintance	-0.08	0.06	0.19	[-0.20, 0.04]
Relative/friend	-0.02	0.06	0.73	[-0.14, 0.10]
Desire for antibiotics (Base = Indicating not wanting antibiotics unless necessary)				
No expression of want	0.32	0.06	< 0.001	[0.20, 0.44]
Indicating wanting antibiotics	0.70	0.11	< 0.001	[0.46, 0.90]
Random effect (Indicating wanting antibiotics)	0.52	0.09	< 0.001	[0.33, 0.70]
Out of pocket payment for medicines (Base = Indicating a maximal out of pocket payment of 30 CNY)				
Willing to paid for all medicines out of pocket	0.05	0.06	0.42	[-0.07, 0.17]
Expense of medicines partly reimbursed by health insurance	-0.03	0.06	0.67	[-0.14, 0.09]
Prescription filling (Base = Outside of the primary care facility)				
Within the primary care facility	0.04	0.04	0.32	[-0.04, 0.12]
Interaction terms				
> 75 years * General Practice	-0.13	0.20	0.51	[-0.52, 0.26]
6 days * General Practice	-0.03	0.16	0.85	[-0.34, 0.28]
9 days * General Practice	0.03	0.27	0.91	[-0.50, 0.57]
Indicating wanting antibiotics * General Practice	-0.10	0.16	0.56	[-0.42, 0.23]
> 75 years* Surgical	0.29	0.24	0.23	[-0.19, 0.76]
6 days * Surgical	-0.07	0.20	0.72	[-0.45, 0.31]
9 days * Surgical	**-0.81**	**0.33**	**0.01**	**[-1.45, -0.17]**
Indicating wanting antibiotics * Surgical	-0.17	0.20	0.39	[-0.55, 0.22]
> 75 years*Others	-0.11	0.17	0.52	[-0.45, 0.23]
6 days * Others	-0.22	0.14	0.12	[-0.49, 0.54]
9 days * Others	**-0.63**	**0.23**	**0.01**	**[-1.09, -0.18]**
Indicating wanting antibiotics * Others	-0.12	0.14	0.39	[-0.40, 0.16]
Model diagnostics				
Number of respondents		386		
Number of observations		9166		

Log Likelihood	-2369.33
Akaike information criterion	4794.66
Bayesian information criterion	4994.11

CNY - Chinese Yuan; Bold font indicates statistical significance at the 0.05 level

Table 9. Interaction effects model estimation for participated physicians' professional title

Attributes	Levels	Interaction effect model 6 – Professional title			
		β	**Standard Error**	***p***	**95 % Confidence Interval**
Age (Base = Younger than 60 years)					
	60-75 years	0.35	0.07	< 0.001	[0.22, 0.48]
	> 75 years	0.55	0.10	< 0.001	[0.36, 0.75]
	Random effect (> 75 years)	0.96	0.08	< 0.001	[0.80, 1.13]
Duration of symptoms (Base = 3 days)					
	6 days	1.04	0.08	< 0.001	[0.88, 1.19]
	Random effect (6 days)	0.30	0.15	0.05	[0.00, 0.60]
	9 days	2.29	0.14	< 0.001	[2.01, 2.57]
	Random effect (9 days)	1.38	0.12	< 0.001	[1.15, 1.61]
Follow-up appointment (Base = Difficult to schedule)					
	Easy to schedule	-0.19	0.04	< 0.001	[-0.28, -0.11]
Familiarity (Base = Stranger)					
	Acquaintance	-0.07	0.06	0.21	[-0.19, 0.04]
	Relative/friend	-0.02	0.06	0.77	[-0.14, 0.10]
Desire for antibiotics (Base = Indicating not wanting antibiotics unless necessary)					
	No expression of want	0.32	0.06	< 0.001	[0.20, 0.44]
	Indicating wanting antibiotics	0.64	0.09	< 0.001	[0.48, 0.81]
	Random effect (Indicating wanting antibiotics)	0.52	0.09	< 0.001	[0.34, 0.70]
Out of pocket payment for medicines (Base = Indicating a maximal out of pocket payment of 30 CNY)					
	Willing to paid for all medicines out of pocket	0.05	0.06	0.46	[-0.08, 0.16]
	Expense of medicines partly reimbursed by health insurance	-0.03	0.06	0.64	[-0.15, 0.09]
Prescription filling (Base = Outside of the primary care facility)					
	Within the primary care facility	0.04	0.04	0.33	[-0.04, 0.12]
Interaction terms					
> 75 years * Middle title		-0.04	0.15	0.80	[-0.33, 0.26]
6 days * Middle title		-0.02	0.12	0.87	[-0.25, 0.21]
9 days * Middle title		-0.21	0.20	0.30	[-0.61, 0.19]
Indicating wanting antibiotics * Middle title		-0.11	0.12	0.35	[-0.35, 0.12]
> 75 years * Vice-senior title		-0.16	0.33	0.64	[-0.81, 0.50]

6 days * Vice-senior title	-0.25	0.26	0.33	[-0.75, 0.25]
9 days * Vice-senior title	0.33	0.46	0.47	[-0.56, 1.22]
Indicating wanting antibiotics * Vice-senior title	-0.02	0.27	0.94	[-0.55, 0.50]
> 75 years * Senior title	-0.70	0.65	0.28	[-1.98, 0.58]
6 days * Senior title	-0.21	0.54	0.70	[-1.27, 0.85]
9 days * Senior title	-0.86	0.86	0.32	[-2.55, 0.83]
Indicating wanting antibiotics * Senior title	-0.57	0.53	0.28	[-1.61, 0.67]
Model diagnostics				
Number of respondents	386			
Number of observations	9166			
Log Likelihood	-2374.86			
Akaike information criterion	4805.72			
Bayesian information criterion	5005.17			

CNY - Chinese Yuan;

Table 10. Interaction effects model estimation for participated physicians' annual household income

Attributes	Levels	Interaction effect model 7 –Annual household income			
		β	**Standard Error**	***p***	**95 % Confidence Interval**
Age (Base = Younger than 60 years)					
	60-75 years	0.35	0.07	< 0.001	[0.22, 0.48]
	> 75 years	0.55	0.14	< 0.001	[0.28, 0.82]
	Random effect (> 75 years)	0.97	0.08	< 0.001	[0.81, 1.14]
Duration of symptoms (Base = 3 days)					
	6 days	0.88	0.11	< 0.001	[0.67, 1.09]
	Random effect (6 days)	0.31	0.15	0.04	[0.02, 0.59]
	9 days	1.87	0.18	< 0.001	[1.52, 2.23]
	Random effect (9 days)	0.51	0.09	< 0.001	[0.33, 0.70]
Follow-up appointment (Base = Difficult to schedule)					
	Easy to schedule	-0.19	0.04	< 0.001	[-0.28, -0.11]
Familiarity (Base = Stranger)					
	Acquaintance	-0.07	0.06	0.24	[-0.19, 0.05]
	Relative/friend	-0.02	0.06	0.77	[-0.14, 0.10]
Desire for antibiotics (Base = Indicating not wanting antibiotics unless necessary)					
	No expression of want	0.32	0.06	< 0.001	[0.21, 0.44]
	Indicating wanting antibiotics	0.64	0.09	< 0.001	[0.48, 0.81]

	Random effect (Indicating wanting antibiotics)	0.53	0.11	< 0.001	[0.31, 0.74]
Out of pocket payment for medicines (Base = Indicating a maximal out of pocket payment of 30 CNY)					
	Willing to paid for all medicines out of pocket	0.05	0.06	0.46	[-0.08, 0.17]
	Expense of medicines partly reimbursed by health insurance	-0.03	0.06	0.65	[-0.14, 0.09]
Prescription filling (Base = Outside of the primary care facility)					
	Within the primary care facility	0.04	0.04	0.35	[-0.04, 0.12]
Interaction terms					
> 75 years * 40,000- 59,999 CNY		-0.01	0.18	0.97	[-0.36, 0.34]
6 days * 40,000- 59,999 CNY		**0.33**	**0.14**	**0.02**	**[0.05, 0.61]**
9 days * 40,000- 59,999 CNY		**0.73**	**0.24**	**< 0.01**	**[0.26, 1.21]**
Indicating wanting antibiotics * 40,000- 59,999 CNY		0.18	0.14	0.21	[-0.10, 0.46]
> 75 years * 60,000- 79,999 CNY		-0.13	0.22	0.56	[-0.55, 0.30]
6 days * 60,000- 79,999 CNY		0.08	0.17	0.62	[-0.25, 0.42]
9 days * 60,000- 79,999 CNY		0.35	0.29	0.23	[-0.22, 0.92]
Indicating wanting antibiotics * 60,000- 79,999 CNY		0.01	0.17	0.94	[-0.32, 0.35]
> 75 years * 80,000- 99,999 CNY		-0.09	0.25	0.71	[-0.59, 0.40]
6 days * 80,000- 99,999 CNY		0.21	0.20	0.29	[-0.18, 0.61]
9 days * 80,000- 99,999 CNY		0.33	0.34	0.33	[-0.33, 0.99]
Indicating wanting antibiotics * 80,000- 99,999 CNY		0.23	0.20	0.25	[-0.17, 0.63]
> 75 years * ⩾100,000 CNY		0.12	0.29	0.67	[-0.44, 0.68]
6 days * ⩾100,000 CNY		-0.17	0.22	0.46	[-0.61, 0.27]
9 days * ⩾100,000 CNY		-0.07	0.39	0.87	[-0.82, 0.69]
Indicating wanting antibiotics * ⩾100,000 CNY		-0.26	0.23	0.26	[-0.71, 0.19]
Model diagnostics					
	Number of respondents		386		
	Number of observations		9166		
	Log Likelihood		-2368.48		
	Akaike information criterion		4800.97		
	Bayesian information criterion		5028.91		

CNY - Chinese Yuan; Bold font indicates statistical significance at the 0.05 level

Table 11. Interaction effects model estimation for participated physicians' educational attainment

Attributes	Levels	Interaction effect model 8 – Educational attainment			
		β	**Standard Error**	***p***	**95 % Confidence Interval**
Age (Base = Younger than 60 years)					
	60-75 years	0.35	0.07	< 0.001	[0.22, 0.48]

> 75 years	0.47	0.19	0.01	[0.10, 0.84]
Random effect (> 75 years)	0.97	0.08	< 0.001	[0.80, 1.13]
Duration of symptoms (Base = 3 days)				
6 days	0.69	0.15	< 0.001	[0.41,0.98]
Random effect (6 days)	0.30	0.15	0.05	[-0.01, 0.60]
9 days	1.90	0.26	< 0.001	[1.39, 2.41]
Random effect (9 days)	1.37	0.12	< 0.001	[1.15, 1.60]
Follow-up appointment (Base = Difficult to schedule)				
Easy to schedule	-0.19	0.04	< 0.001	[-0.28, -0.11]
Familiarity (Base = Stranger)				
Acquaintance	-0.07	0.06	0.22	[-0.19, 0.04]
Relative/friend	-0.02	0.06	0.75	[-0.14, 0.10]
Desire for antibiotics (Base = Indicating not wanting antibiotics unless necessary)				
No expression of want	0.32	0.06	< 0.001	[0.21, 0.44]
Indicating wanting antibiotics	0.42	0.15	< 0.001	[0.12, 1.72]
Random effect (Indicating wanting antibiotics)	0.52	0.09	< 0.001	[0.34, 0.70]
Out of pocket payment for medicines (Base = Indicating a maximal out of pocket payment of 30 CNY)				
Willing to paid for all medicines out of pocket	0.05	0.06	0.46	[-0.07, 0.17]
Expense of medicines partly reimbursed by health insurance	-0.03	0.06	0.64	[-0.15, 0.09]
Prescription filling (Base = Outside of the primary care facility)				
Within the primary care facility	0.04	0.04	0.34	[-0.04, 0.12]
Interaction terms				
> 75 years * Associate degree	-0.03	0.21	0.87	[-0.45, 0.38]
6 days * Associate degree	0.32	0.16	0.05	[-0.01, 0.65]
9 days * Associate degree	0.14	0.28	0.63	[-0.42, 0.70]
Indicating wanting antibiotics * Associate degree	0.14	0.17	0.41	[-0.19, 0.47]
> 75 years * University degree	0.20	0.22	0.36	[-0.23, 0.64]
6 days * University degree	**0.46**	**0.17**	**0.01**	**[0.11, 0.80]**
9 days * University degree	**0.71**	**0.30**	**0.02**	**[0.12, 1.30]**
Indicating wanting antibiotics * University degree	0.31	0.18	0.08	[-0.04, 0.66]
Model diagnostics				
Number of respondents		386		
Number of observations		9166		
Log Likelihood		-2369.84		
Akaike information criterion		4787.67		
Bayesian information criterion		4958.63		

CNY - Chinese Yuan; Bold font indicates statistical significance at the 0.05 level

Table 12. Interaction effects model estimation for whether receiving educational materials on antibiotic prescribing

Attributes	Levels	Interaction effect model 9 – Whether receiving educational materials on antibiotic prescribing			
		β	**Standard Error**	***p***	**95 % Confidence Interval**
Age (Base = Younger than 60 years)					
	60-75 years	0.34	0.07	< 0.001	[0.21, 0.47]
	> 75 years	0.63	0.97	0.52	[-1.27, 2.53]
	Random effect (> 75 years)	0.97	0.08	< 0.001	[0.81, 1.13]
Duration of symptoms (Base = 3 days)					
	6 days	1.53	0.68	0.02	[0.20,2.86]
	Random effect (6 days)	0.32	0.14	0.03	[0.04, 0.60]
	9 days	3.99	1.41	0.01	[1.23, 6.76]
	Random effect (9 days)	1.39	0.12	< 0.001	[1.17, 1.62]
Follow-up appointment (Base = Difficult to schedule)					
	Easy to schedule	-0.19	0.04	< 0.001	[-0.28, -0.11]
Familiarity (Base = Stranger)					
	Acquaintance	-0.08	0.06	0.21	[-0.19, 0.04]
	Relative/friend	-0.02	0.06	0.75	[-0.14, 0.10]
Desire for antibiotics (Base = Indicating not wanting antibiotics unless necessary)					
	No expression of want	0.32	0.06	< 0.001	[0.20, 0.44]
	Indicating wanting antibiotics	0.69	0.79	0.39	[-0.87, 2.24]
	Random effect (Indicating wanting antibiotics)	0.52	0.09	< 0.001	[0.34, 0.71]
Out of pocket payment for medicines (Base = Indicating a maximal out of pocket payment of 30 CNY)					
	Willing to paid for all medicines out of pocket	0.05	0.06	0.46	[-0.07, 0.17]
	Expense of medicines partly reimbursed by health insurance	-0.03	0.06	0.63	[-0.15, 0.09]
Prescription filling (Base = Outside of the primary care facility)					
	Within the primary care facility	0.04	0.04	0.31	[-0.04, 0.13]
Interaction terms					
> 75 years * Not sure		-0.24	1.03	0.82	[-2.26, 1.79]
6 days * Not sure		-0.59	0.74	0.42	[-2.04, 0.85]
9 days * Not sure		-1.51	1.49	0.31	[-4.44, 1.42]
Indicating wanting antibiotics * Not sure		0.12	0.85	0.89	[-1.54, 1.78]
> 75 years * Yes		-0.11	0.97	0.91	[-2.01, 1.80]
6 days * Yes		-0.52	0.68	0.45	[-1.85, 0.82]
9 days * Yes		-1.79	1.41	0.21	[-4.60, 0.98]
Indicating wanting antibiotics * Yes		-0.10	0.80	0.90	[-1.66, 1.46]

Model diagnostics	
Number of respondents	386
Number of observations	9166
Log Likelihood	-2377.14
Akaike information criterion	4802.29
Bayesian information criterion	4973.24

CNY - Chinese Yuan;

Table 13. Interaction effects model estimation for whether attending antibiotic training course over the past year

**Attributes**	**Levels**	**Interaction effect model 10 – Whether attending antibiotic training course over the past year**			
		β	**Standard Error**	***p***	**95 % Confidence Interval**
Age (Base = Younger than 60 years)					
	60-75 years	0.34	0.07	< 0.001	[0.21, 0.47]
	> 75 years	0.31	0.20	0.12	[-0.08, 0.70]
	Random effect (> 75 years)	0.97	0.08	< 0.001	[0.81, 1.13]
Duration of symptoms (Base = 3 days)					
	6 days	1.00	0.16	< 0.001	[0.69, 1.31]
	Random effect (6 days)	0.32	0.14	0.03	[0.04, 0.60]
	9 days	2.07	0.27	< 0.001	[1.54, 2.61]
	Random effect (9 days)	1.40	0.12	< 0.001	[1.17, 1.62]
Follow-up appointment (Base = Difficult to schedule)					
	Easy to schedule	-0.19	0.04	< 0.001	[-0.28, -0.11]
Familiarity (Base = Stranger)					
	Acquaintance	-0.08	0.06	0.22	[-0.19, 0.04]
	Relative/friend	-0.02	0.06	0.76	[-0.14, 0.10]
Desire for antibiotics (Base = Indicating not wanting antibiotics unless necessary)					
	No expression of want	0.32	0.06	< 0.001	[0.20, 0.44]
	Indicating wanting antibiotics	0.57	0.16	< 0.001	[0.25, 0.89]
	Random effect (Indicating wanting antibiotics)	0.52	0.09	< 0.001	[0.34, 0.70]
Out of pocket payment for medicines (Base = Indicating a maximal out of pocket payment of 30 CNY)					
	Willing to paid for all medicines out of pocket	0.05	0.06	0.46	[-0.07, 0.17]
	Expense of medicines partly reimbursed by health insurance	-0.03	0.06	0.63	[-0.15, 0.09]
Prescription filling (Base = Outside of the primary care facility)					
	Within the primary care facility	0.04	0.04	0.33	[-0.04, 0.12]
Interaction terms					
> 75 years * Not sure		0.07	0.48	0.88	[-0.87, 1.02]

6 days * Not sure	-0.05	0.40	0.90	[-0.83, 0.72]
9 days * Not sure	-0.29	0.67	0.66	[-1.60, 1.01]
Indicating wanting antibiotics * Not sure	-0.05	0.39	0.90	[-0.82, 0.72]
> 75 years * Yes	0.25	0.21	0.24	[-0.17, 0.66]
6 days * Yes	0.03	0.17	0.88	[-0.31, 0.36]
9 days * Yes	0.19	0.29	0.50	[-0.37, 0.76]
Indicating wanting antibiotics * Yes	0.04	0.17	0.84	[-0.30, 0.37]
Model diagnostics				
Number of respondents	386			
Number of observations	9166			
Log Likelihood	-2377.54			
Akaike information criterion	4803.08			
Bayesian information criterion	4974.04			

CNY - Chinese Yuan;
